# Effects of Sublethal Organophosphate Toxicity and Anti-cholinergics on Electroencephalogram and Respiratory Mechanics in Mice

**DOI:** 10.3389/fnins.2022.866899

**Published:** 2022-05-02

**Authors:** Vladislav Bugay, Summer Rain Gregory, Matthieu Gibson Belanger-Coast, Raymond Zhao, Robert Brenner

**Affiliations:** Department of Cellular and Integrative Physiology, University of Texas Health San Antonio, San Antonio, TX, United States

**Keywords:** organophosphate, seizure, respiration, EEG, anticholinergic

## Abstract

Organophosphates are used in agriculture as insecticides but are potentially toxic to humans when exposed at high concentrations. The mechanism of toxicity is through antagonism of acetylcholinesterase, which secondarily causes excess activation of cholinergic receptors leading to seizures, tremors, respiratory depression, and other physiological consequences. Here we investigated two of the major pathophysiological effects, seizures and respiratory depression, using subcutaneous injection into mice of the organophosphate diisopropylfluorophosphate (DFP) at sublethal concentrations (2.1 mg/Kg) alone and co-injected with current therapeutics atropine (50 mg/Kg) or acetylcholinesterase reactivator HI6 (3 mg/Kg). We also tested a non-specific cholinergic antagonist dequalinium chloride (2 mg/Kg) as a novel treatment for organophosphate toxicity. Electroencephalogram (EEG) recordings revealed that DFP causes focal delta frequency (average 1.4 Hz) tonic spikes in the parietal region that occur transiently (lasting an average of 171 ± 33 min) and a more sustained generalized theta frequency depression in both parietal and frontal electrode that did not recover the following 24 h. DFP also caused behavioral tremors that partially recovered the following 24 h. Using whole body plethysmography, DFP revealed acute respiratory depression, including reduced breathing rates and tidal volumes, that partially recover the following day. Among therapeutic treatments, dequalinium chloride had the most potent effect on all physiological parameters by reducing acute EEG abnormalities and promoting a full recovery after 24 h from tremors and respiratory depression. Atropine and HI6 had distinct effects on EEGs. Co-treatment with atropine converted the acute 1.4 Hz tonic spikes to 3 Hz tonic spikes in the parietal electrode and promoted a partial recovery after 24 h from theta frequency and respiratory depression. HI6 fully removed the parietal delta spike increase and promoted a full recovery in theta frequency and respiratory depression. In summary, while all anticholinergic treatments promoted survival and moderated symptoms of DFP toxicity, the non-selective anti-cholinergic dequalinium chloride had the most potent therapeutic effects in reducing EEG abnormalities, moderating tremors and reducing respiratory depression.

## Introduction

Organophosphates are the main components of pesticides and insecticides. Organophosphates act primarily by irreversible inhibition of acetylcholinesterase enzymes. Organophosphates have been useful in controlling insects both in agriculture and for abatement of disease vectors such as mosquitos (Raghavendra et al., 2011; Stoops et al., 2019). Due to their widespread use, toxic levels of organophosphate exposure in humans are a common cause of poisoning (Gummin et al., 2020). Organophosphates have also been coopted for use as chemical weapons (Sidell and Borak, 1992; Evison et al., 2002). Organophosphate inhibition of acetylcholinesterase results in excess acetylcholine in synapses and symptoms associated with hyper-activation of cholinergic receptors (Atchison, 1988). There are numerous health effects of organophosphate toxicity that are physiologically related to activation of peripheral muscarinic and nicotinic acetylcholine receptors. These include bronchoconstriction and bronchorrhea, muscle tremors and fasciculations, bradycardia, and vomiting (Alozi and Rawas-Qalaji, 2020; Aman et al., 2021). Central nervous system effects include respiratory depression, depressed consciousness, and seizures (Reji et al., 2016; Williamson et al., 2019).

Current mainstay of therapy for organophosphate poisoning is the muscarinic acetylcholine receptor antagonist atropine. Antagonism of muscarinic receptors is known to decrease exocrine and respiratory secretions, reduce bronchospasms, prevent bradycardia, and protect against cholinergic seizures (Carpentier et al., 2000; Aman et al., 2021). In addition, therapeutics that directly target organophosphate mechanisms are reactivators of acetylcholinesterase. These include the cationic oximes HI6 [1-([2-([*E*]-hydroxyiminomethyl)pyridin-1-ium-1-yl]methoxymethyl)pyridin-1-ium-4-carboxamide] and 2-PAM (pralidoxime) (Kobrlova et al., 2019; Worek et al., 2020). Given that inhibition of acetylcholinesterase promotes activation of both muscarinic and nicotinic acetylcholine receptors, it is logical that antidote therapy could be improved by also including inhibitors of nicotinic receptors. Indeed, symptoms of organophosphate toxicity, including tremors (Iha et al., 2019) and muscle weakness (Bird et al., 2016), have been ameliorated by drugs that specifically target nicotinic acetylcholine receptor activation (Bird et al., 2016; Iha et al., 2019).

Given that pharmacological antagonism of both nicotinic and muscarinic receptor types has been shown to protect against organophosphate toxicity (Chiou et al., 1986; Rosenbaum and Bird, 2010; Williamson et al., 2019), a logical therapeutic strategy is to use non-selective cholinergic antagonists. Here we wished to investigate the non-selective anticholinergic dequalinium chloride, a hydrophobic compound first described as an anti-bacterial agent (Babbs et al., 1956) and oral disinfectant mouthwash (i.e., Dequadin and others) (Kaufman, 1981; Griffiths and Stock, 1986; Liberman et al., 1989). Dequalinium was later shown to block cholinergic neurotransmission responses to nicotinic receptor receptors both in neuronal synapses (Dunn, 1993) and at the neuromuscular junction, while also proving to be an effective blocker of SK potassium channels (Castle et al., 1993; Dunn, 1994). Dequalinium was recently shown to antagonize muscarinic receptor activation of all subtypes (Bugay et al., 2020b; Mazzolari et al., 2020) as well.

Our approach to evaluate treatments for organophosphate toxicity was to first characterize a range of critical physiological parameters affected by organophosphates, before comparing the effects of established therapeutics to novel treatments. Here we used diisopropylfluorophosphate (DFP), a common model for organophosphate toxicity, employing identical dosages and timelines to examine both neurological effects and respiratory mechanics. We studied sublethal concentrations of organophosphate, which better represent most toxic events (Muley et al., 2014). We also used this experimental paradigm to examine therapeutic effects of common treatments atropine and HI6 and investigate the non-selective cholinergic antagonist dequalinium chloride as a novel treatment for organophosphate toxicity.

## Materials and Methods

### Animals

Both male and female adult (8–12 weeks old) C57BL/6J mice were used. Mice were group-housed in a 12-h light-dark cycle with food and water *ad libitum*. All experiments were undertaken in accordance with Institutional Animal Care and Use Committee at the University of Texas Health San Antonio (UT Health SA, protocol 20210002AP) and were in compliance with the National Institutes of Health *Guide for Care and Use of Laboratory Animals*. The experiments were conducted using two groups of mice. The first group were implanted with electroencephalogram (EEG) headmounts and coincidently assessed for effects on abnormal EEG waveforms and behavioral tremors using coincident video-EEG recordings. A second group of mice (without EEG headmounts) were assessed for effects on respiratory function using unrestrained whole animal plethysmography. The combined two groups were used to assay lethality.

### Diisopropylfluorophosphate Exposures and Drug Treatments

Stock solutions of diisopropylfluorophosphate (DFP, 1.06 g/ml density, Sigma D0879) were diluted with PBS (1 μL in 4 mL PBS, 0.265 mg/ml of DFP) and vortexed for 1 min before use. The DFP solution was injected into mice subcutaneously (sc) in the right pelvic area at a volume of 8 μl per gram weight, resulting in a dosage of 2.1 mg/Kg that caused 33% (8/24) lethality without therapeutic cotreatment. A slightly higher concentration of 2.8 mg/Kg was found to cause 80% lethality (8/10) and was not used. Control saline or treatments were injected sc immediately after DFP (within 30 s) in the left pelvic area at 8 μl per gram weight to obtain concentrations of 50 mg/Kg for HI6 (Sigma SML0224), 3 mg/Kg of Atropine sulfate (Sigma A0257) and 2 mg/Kg of dequalinium chloride (Sigma PHR1300). Injections were conducted under transient isoflurane gas anesthesia. All treatment compounds were dissolved as stock solutions in sterile water and stored frozen except dequalinium chloride, which required dissolution in 100% methanol at 5 mM (2.6 mg/ml) and further dilution into saline on the day of the experiment. It is worth noting that unlike other reports (Manetta et al., 1993; Yu et al., 2020) we found that dequalinium chloride did not dissolve in aqueous solutions (even with heating and sonication).

### Pilocarpine-Induced Seizure

Thirty minutes prior to administration of pilocarpine (300 mg/kg, Tocris Bioscience, Bristol, United Kingdom) EEG-implanted mice received an injection of scopolamine methylnitrate 1 mg/kg (MP Biomedicals, Santa Ana, CA, United States) that does not cross the blood-brain barrier and inhibits peripheral pilocarpine effects. Pilocarpine was delivered via intraperitoneal (I.P.) injection in 0.9% normal saline (pH 7.4). The mice were confirmed to have electrographic and behavioral seizure activity within 30 min following injection.

### Electroencephalogram Recording

Mice were implanted with electroencephalogram (EEG) screw-type electrodes under general anesthesia using inhaled isoflurane (5%). A tethered EEG system with a prefabricated head-mount containing the screw electrodes was used (catalog number 8201-EEG; Pinnacle Technology, Inc., Lawrence, Kansas, United States). The head-mount included a ground screw (left frontal), and a reference screw electrode (left parietal) for right frontal (F, channel 1) and the right parietal (P, channel 2) electrodes as previously described (Bugay et al., 2020a). The recording electrodes were implanted at positions + 1.4, 2.3 mm (frontal) and + 1.4, −3.7 mm (parietal) relative to medial and bregma, respectively. Postoperatively, animals were housed individually and carefully monitored for pain and distress. Mice recovered from surgery a minimum of 48 h before exposure to DFP or treatments.

For video-EEG recordings, the head-mount was connected to a pre-amplifier (Gain 25X; Pinnacle Technologies), which in turn was connected via wires to a commutator and extracellular amplifiers of the Stellate Harmonie acquisition hardware. The system collected coincident video images of the animals during EEG activity. The EEG records were scored using a 0.1–35 Hz bandpass filter and a 60 Hz notch noise filter. Preceding chemical exposures, mice were recorded for a minimum of 2 h before they were removed and injected under transient isoflurane anesthesia with DFP alone or with treatments at dosages described above. The mice were quickly returned to the recording chamber and monitored for an additional 24 h. Brain activity was assayed visually by the investigator and also by the computer-assisted EEG stellate harmonie review software.

### Power Analysis of Electroencephalogram Records

For each experiment, a period of 1-h EEG recording before drug injection was used as a control. A period of 1-h EEG after drug injection was used for analyzing the drug’s acute effects and after 23-h EEG recording the 1-h EEG period was used for analyzing recovery. After visual inspection of the video-EEGs, 4-s epochs EEG power spectra were calculated by Fast Fourier Transform (FFT). FFT lengths of 5.1 s and Spectral Restart Intervals of 10.24 s were used. Mean square root power values (μV/Hz) were then normalized to the total power to obtain relative values comparable among the different animals.

Statistical significance was determined depending on the experimental design. The unpaired *t*-test, one-way or two-way ANOVA with repeated measures followed by Tukey’s multiple comparison test were performed using GraphPad Prism 9.0. All data are expressed as mean ± standard error of the mean (SEM). Statistical significance was considered at *p* < 0.05.

### Behavioral Assays of Tremors

**FIGURE 1:**
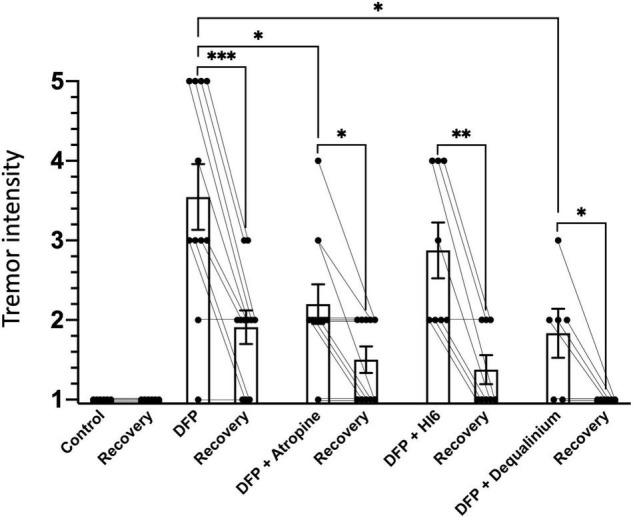
Tremor intensity in response to diisopropylfluorophosphate (DFP) alone or DFP with treatments. Bars indicate average tremor intensity and standard error of means for 1 h immediately after injection (1st bar), and 22–24 h after injection (recovery, 2nd bar). The number of animals scored for each group were: control (saline), 6; DFP alone, 11; DFP with atropine, 10; DFP with HI6, 8; and DFP with dequalinium chloride, 6. Statistical significance was determined with one-way ANOVA between treatment groups, and paired t-test to compare acute tremors and recovery within a treatment group. Significance of *p* < 0.05, *p* < 0.01, *p* < 0.001 is indicated with single, double, and triple asterisks, respectively.

To assay tremors, an experimenter blinded to the treatment performed the behavioral classification. Visual inspection of the video recordings (removed of EEG data) for the 1 h period following injection of DFP alone or DFP with treatments were scored. Animals were scored 1 if no tremors were apparent, 2 if rare tremors were apparent, 3 if tremors occurred approximately through 50% of the recording period, 4 if the mice had rare periods without tremors, and 5 if tremors occurred continuously. It is worth noting that control animals having vehicle injections (Figure 1) or anticholinergics in the absence of DFP (data not shown) had no detectable tremors. The mice were not scored on a Racine scale since we did not see any other behaviors that are typical of seizures such as rearing and falling, clonus, head bobbing, running or jumping.

### Plethysmography

Unrestrained whole animal plethysmography of mice was conducted using the BUXCO system (Buxco Research Systems, Wilmington, NC, United States) according to previous procedures (Lomask, 2006). Plethysmography chambers were calibrated for 1 ml of flow preceding each experiment. Mice were allowed 60 min to acclimate to the chambers. For chemical exposures, the mice were removed from the chambers and injected with DFP alone or DFP with treatments as described above. Injections were conducted during acute isoflurane anesthesia, where the mice recovered approximately 20–30 s after chemical injections before returning to the plethysmography chambers. The mice were recorded for an additional 1 h following injections and then for 1 h the following 22–24 h after injections to assay recovery.

## Results

### Survival to Diisopropylfluorophosphate Alone and With Treatments

In order to understand the neuronal and respiratory pathophysiological effects of organophosphate toxicity and their therapeutic interventions, we employed a concentration of DFP that was below the LD_50_ (2.1 mg/Kg, 0.64 LD_50_) based on previous studies (Tripathi and Dewey, 1989). This allowed us to study both acute (<1 h) and sustained effects (22–24 h) during the recovery period. Using this concentration, we found that 67% (16/24) of animals survived 24 h after DFP exposure. In contrast, standard treatments (Hulse et al., 2019; Alozi and Rawas-Qalaji, 2020; Aman et al., 2021) delivered coincident with DFP almost fully eliminated lethality. This included the muscarinic antagonist Atropine (3 mg/Kg), which promoted 94% (16/17) survival, and the acetylcholinesterase reactivator HI6 (50 mg/Kg), which caused 100% (16/16) survival. We also tested a non-specific anticholinergic, dequalinium chloride (2 mg/Kg), which antagonizes both muscarinic (Bugay et al., 2020b) and nicotinic receptor activation (Dunn, 1993). It was 100% effective (11/11 survived) in eliminating lethality.

### Effect on Diisopropylfluorophosphate-Induced Tremors

Tremors and involuntary or uncontrollable movements are an obvious symptom of organophosphate poisoning (Reji et al., 2016). Behavioral scoring was used to quantify tremor severity (scored in a range from 1 for no tremors to 5 with constant tremors). In the absence of DFP, an observer blind to treatments scored all animals 1 for severity (Figure 1). Treatment with DFP revealed obvious tremors during the first hour of exposure (Figure 1, tremor score 3.5 ± 0.41). The severity of tremors declined following 22–24 h of recovery (Figure 1, 1.91 ± 0.21, *P* = 0.0004), but was still elevated above control levels (*P* = 0.007). Both atropine (2.2 ± 0.28, *P* = 0.014) and dequalinium chloride (1.8 ± 0.30, *P* = 0.013), but not HI6 (2.9 ± 0.35, *P* = 0.52), significantly reduced DFP tremors within 1 h following DFP exposure (Figure 1). During the recovery phase (22–24 h after exposure), dequalinium chloride (1.0 ± 0, *P* = 0.007), but not atropine (1.5 ± 0.16, *P* = 0.24) or HI6 (1.4 ± 0.52, *P* = 0.11), showed significantly better recovery than recovery following DFP alone exposure (Figure 1).

### Effect of Diisopropylfluorophosphate on Electroencephalogram Activity

**FIGURE 2:**
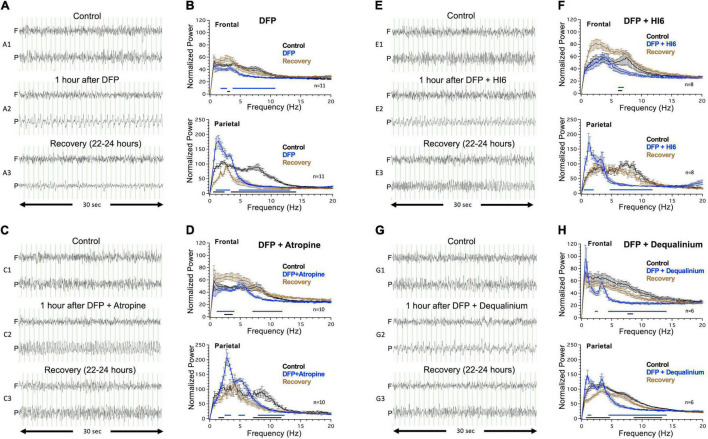
Electroencephalogram (EEG) activity in response to injections of diisopropylfluorophosphate (DFP) alone or DFP with treatment. Left panels show example traces before (top traces, **A1–G1**), immediately after (middle traces, **A2–G2**), and 22–24 h after DFP or DFP with treatment (bottom traces, **A3–G3**). Traces are shown for frontal (F) and parietal electrodes (P). Vertical grid lines represent 1 s intervals. Right panels display power analysis across a 15 min sampling period that includes the representative example traces. Power analysis are for control records (black), records immediately following DFP alone or DFP with treatment (blue), or recovery 22–24 h after injection (brown). Right top panel show analysis from frontal electrode 1, and right bottom panel show analysis for parietal electrode 2. Lines below the graph are regions where there was a statistically significant difference (Two-way ANOVA, Tukey multiple comparisons test, *P* < 0.05) in normalized power between control and acute EEG response (blue line), or control and recovery (black line). Data is for analysis of 11 mice for DFP alone, 10 mice for DFP with atropine, eight mice for DFP with HI6, and six mice for DFP with dequalinium chloride.

Electroencephalogram records across three time periods were analyzed: a control 1 h period immediately before, 1 h immediately after subcutaneous DFP injection (2.1 mg/Kg), and a 1 h period at 22–24 h after DFP injection (recovery period). Example records are shown in Figure 2A with traces for frontal (F) and parietal electrodes (P) taken before (Figure 2A1), immediately following (Figure 2A2), and 22–24 h after injection of DFP (Figure 2A3). Before DFP injections, the EEG traces at the frontal and parietal electrodes are very similar and show normal activity (Figure 2A1). For the frontal electrode, DFP had little effect by visual inspection (F electrode, Figure 2A2). For the parietal electrode, DFP injection (P, Figure 2A2) caused a prominent and sustained synchronization of cortical activity of ∼1–2 Hz tonic spikes. The focal spikes, although abnormal and rhythmic, do not show the evolution of amplitude and frequency seen of typical electrographic seizure activity of other chemoconvulsants (Fisher et al., 2014; Supplementary Figure 1). The EEG pattern was apparent for long durations (average duration of 171 ± 33 min per mouse, *N* = 11). For periods where abnormal electrographic activity was or was not present, there was no correlation with tremor behavior. The following day, the 1–2 Hz tonic spikes pattern seen in the parietal electrode had dissipated, although the EEG trace showed significantly reduced amplitudes (Figure 2A3). It is worth noting that detection of conventional seizures evoked with muscarinic agonist pilocarpine using the electrodes employed here showed generalized electrographic seizures that appear on both forebrain and caudal parietal electrodes (Supplementary Figure 1). Thus, the focal, parietal-specific spike pattern appears to be unique to the DFP model using sublethal concentrations.

Power spectrum analysis of a 15 min sampling of regions of abnormal activity provides a qualitative assessment of the EEG abnormalities observed during DFP exposure (blue trace, Figure 2B). For comparison, the graph also includes power analysis of the EEGs before (black trace) and 22–24 h following (brown trace) DFP exposure (Figure 2B). Two major changes are clearly discernable in the parietal electrode (Figure 2B, Parietal). DFP causes an enhancement in the delta region (1–4 Hz) spike patterns with highest power at 1.4 Hz that declines the following day after recovery (Figure 2B, Parietal). In addition, there is a dramatic reduction of the theta (4–8 Hz) through alpha range (8–13 Hz) following DFP exposure that is not recovered the following day (Figure 2B, Parietal). In the frontal electrode, acute DFP did not cause an increase in the delta frequencies (Figure 2B, Frontal) but did cause a decrease in power across the 2–11 Hz frequencies that recovered the following day (Figure 2B, Frontal). We also conducted power spectra analysis across a longer 1 h period following DFP injection, which is less biased for abnormal events than a 15 min analysis. The power spectra for DFP indeed shows a significant increase in the 1.4 Hz peak and decline in theta frequencies (Supplementary Figure 2).

### Effect of Organophosphate Treatments on Electroencephalogram Activity

#### Effect of Atropine

Coinjection with muscarinic receptor antagonist atropine (3 mg/Kg) reduced but did not eliminate EEG abnormalities despite its use as a standard of treatment for organophosphate toxicity. Rather than 1.4 Hz spikes caused by DFP alone (Figure 2A), coinjection of atropine caused higher frequency spiking in the parietal electrode (Figure 2C2) that was shifted to a peak 3 Hz frequency in the power analysis (Figure 2D, Parietal). The abnormal high frequency DFP/atropine spiking had an average duration of 125 ± 41 min (*N* = 10), which was on-average reduced from the 171 ± 41 min for DFP alone (*N* = 11) but not statistically different (*P* = 0.39). Similar to DFP alone, DFP with atropine showed a decline in the 7–12 Hz frequencies that was not recovered the following day. As a control, we also injected atropine in the absence of organophosphate and did not see abnormal EEG activity (*N* = 3, Supplementary Figure 2). The following day, mice recovered from the delta frequency oscillations but maintained a depression in the theta frequencies (Figures 2C,D).

#### Effect of HI6

The acetylcholinesterase reactivator HI6 (50 mg/Kg) was also examined. Like DFP alone, mice treated with DFP and HI6 had an increased delta frequency with spikes occurring at 1.3 Hz in the parietal electrode (Figures 2E,F). However, these tended to be shorter lasting (98 ± 42 min) than DFP alone (*P* = 0.18) and were not apparent when included in a 1 h power analysis (Supplementary Figure 2). A key difference from atropine was that HI6 caused a recovery from the depressed theta frequency the following day (Figures 2E,F). Comparison of power spectrum before (black trace) and 22–24 h after DFP with HI6 (brown trace) showed no difference across most of the power spectra (Figure 2F, Parietal). It is worth noting that HI6 treatment alone had slight acute effects on the spectra, causing slight increases at 4–5 Hz and slight decreases at 12–15 Hz (Supplementary Figure 2B).

#### Effect of Dequalinium Chloride

Dequalinium chloride (2 mg/Kg) coinjection with DFP also had enhanced delta frequency components (Figures 2G2,H), but these were significantly reduced (47 ± 21 min, *P* = 0.02) from DFP alone (171 ± 33). Indeed, analysis of the power spectra across 1 h time periods failed to reveal the delta wave component following treatment (Supplementary Figure 2). Like HI6, dequalinium chloride caused a full recovery of the EEG the following day (Figures 2G3,H). As controls, we also investigated the effect of dequalinium alone on the EEG waveforms and did not see any effects (*N* = 3, Supplementary Figure 2).

### Effect on Respiratory Mechanics

Effects of organophosphate toxicity on respiration has been well-documented (Hulse et al., 2014), and indeed respiratory failure is proposed to be a cause of organophosphate mortality (Hulse et al., 2019; Aman et al., 2021). Therefore, we examined the effect of DFP and treatments on mouse respiratory mechanics using identical timelines and drug concentrations as used in the EEG studies. The mice were studied using non-invasive whole body plethysmography, where others have documented effects of organophosphate on respiratory mechanics (Villa et al., 2007; Houze et al., 2008, 2018, 2019). For these studies, we used breathing rate and tidal volumes as reporters for DFP respiratory toxicity.

**FIGURE 3:**
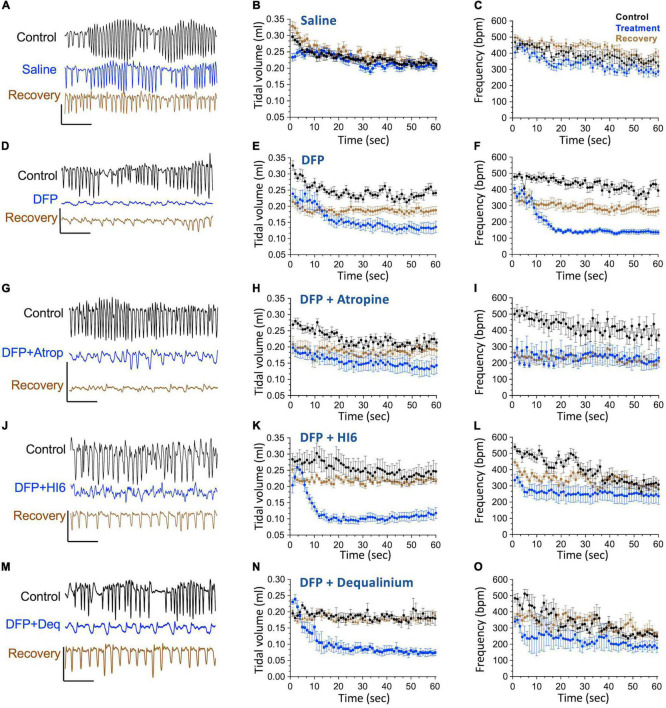
Timeline of breathing frequency (breaths per minute) and tidal volume (ml) in response to diisopropylfluorophosphate (DFP) alone or DFP with treatments. Left panels **(A,D,G,J,M)** show example flow traces for mice before (black), acutely after treatment (treatment indicated, blue) and 22–24 h after treatment (brown, recovery). Middle and right panels show averages of tidal volumes (ml volumes, middle panels) and breathing frequency (breathes per minute, right panels) before (control, black), acutely after treatment (treatment, blue), and 22–24 h after treatment (brown, recovery) for saline treatment **(B,C)**, DFP alone **(E,F)**, DFP with Atropine **(H,I)**, DFP with HI6 **(K,L)**, and DFP with dequalinium chloride **(N,O)**. Data is for analysis of 10 mice for saline alone, 10 mice for DFP alone, seven mice for DFP with atropine, eight mice for DFP with HI6, and four mice for DFP with dequalinium chloride. Scale bars in the left panels indicate 1 ml/s flow (vertical) and 1 s time (horizontal).

**FIGURE 4:**
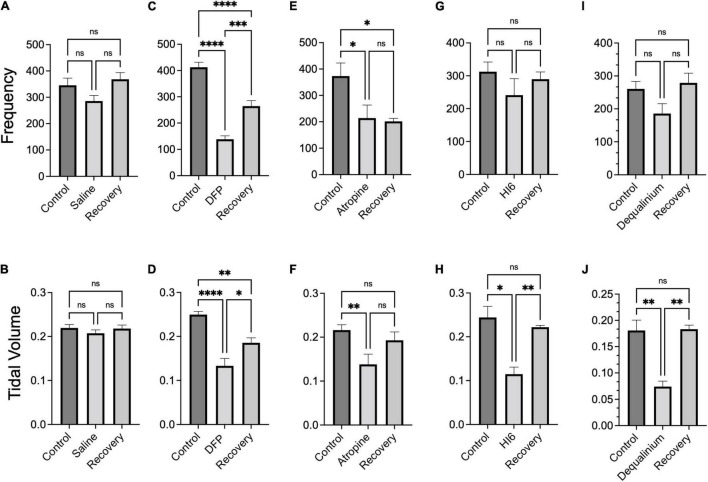
Summary breathing frequency (breaths per minute) and tidal volume (ml) changes in response to diisopropylfluorophosphate (DFP) alone or DFP with treatments. Bar graphs show average breathing frequency (top panels) and tidal volumes (bottom panels) before (control), acutely after DFP or DFP with treatment (middle bar) and 22–24 h after treatment (recovery). Data is the average across the last 5 min of 1 h measurements (data from Figure 3) of breathing frequency and tidal volume, respectively, for saline treatment **(A,B)**, DFP alone treatment **(C,D)**, DFP with Atropine **(E,F)**, DFP with HI6 **(G,H)**, and DFP with dequalinium chloride **(I,J)**. Data is for analysis of 10 mice for saline alone, 10 mice for DFP alone, seven mice for DFP with atropine, eight mice for DFP with HI6, and four mice for DFP with dequalinium chloride. Statistical significance was determined with one-way ANOVA comparison between treatments. Significance of < 0.05, *p* < 0.01, *p* < 0.001 is indicated with single, double, and triple asterisks, respectively.

Similar to EEG studies, we assayed breathing mechanics (Figure 3) for 1 h periods before treatments (control, black traces), immediately after treatments (acute effects, blue traces), and 22–24 h following treatments (recovery phase, brown traces). Preceding DFP injections, control measurements show a decline in breathing rate and tidal volumes as mice acclimate to the chamber (Figures 3A–C). For comparison, we therefore selected the average of the last 5 min of each 1 h period where tidal volumes approach a plateau (Figure 3B). These are plotted as summary data in Figure 4. Transient removal of the mice from the chambers and DFP injection caused a dramatic reduction in tidal volumes and breathing rate (Figures 3D–F, respectively) that was well below values for mice injected with saline alone (Figures 3A–C, summarized in Figures 4C,D). The following day, mice did not fully recover and had depressed tidal volumes and breathing rates that were approximately intermediate to that of the control and DFP injection (Figures 4C,D). Atropine treatment did not rescue acute effects of DFP, although it promoted recovery with regard to tidal volumes (Figure 4F) but not breathing rates (Figure 4E). Both HI6 and dequalinium had the most protective effects on respiratory mechanics. They had acute protective effects with regard to breathing rates, but not tidal volumes, and promoted recovery in both parameters (Figures 4G–J).

As controls, we conducted plethysmography studies for effects of the treatments in the absence of organophosphate (Supplementary Figure 3). In all cases, the treatments alone lacked significant effect during or following recovery relative to pretreatment controls. In order to compare plethysmography findings between treatment groups, we also normalized the plethysmography data to control values before DFP exposure (Supplementary Figure 4). Comparisons between treatments groups (Supplementary Figure 4) yielded results that were consistent to non-normalized data (Figure 4). Both HI6 and dequalinium were significantly improved compared to DFP alone (*p* < 0.05) for breathing frequency acutely and following recovery, and improved recovery of tidal volumes (Supplementary Figure 4). However all treatments were not significantly different than DFP alone for acute effects on tidal volume (Supplementary Figure 4). In summary, although none of the treatments appear to protect fully to acute DFP exposure, some treatments such as HI6 and dequalinium improved recovery of respiration.

## Discussion

Given the broad distribution of cholinergic receptors, it was not surprising that DFP caused numerous effects even at the sublethal concentrations used in this study. These included the physiological parameters measured in this study: tremors, abnormal electrocortical activity, and respiratory depression. One aspect that was unique to this compound was that the abnormal EEGs are different than other chemoconvulsant compounds such as pilocarpine, a non-selective muscarinic acetylcholine receptor agonist, despite the fact that both compounds cause excess activation of muscarinic receptors. Unlike pilocarpine seizures, the abnormal electrographic activity did not evolve during onset, and were apparent as an increase in tonic delta frequency spikes and a depression of theta frequencies. In contrast, the pattern of pilocarpine electrographic seizure onset evolve from low frequency to higher frequency that generalizes to the whole cortex (Curia et al., 2008). The depression of theta frequencies is more reminiscent of pre-ictal, latent period early after pilocarpine exposure (Phelan et al., 2015). Also, despite presumably broad CNS exposure that would be expected from subcutaneous injection, DFP caused some aspects of the abnormal EEG activity to be localized to the caudal parietal region of the brain (delta frequency), while causing other aspects such as the theta frequencies to be depressed in both frontal and parietal electrodes. Others have also observed an increase in delta frequencies and decrease in theta frequencies due to high concentrations of the organophosphate soman (pinacolyl methylfluorophosphonate) (Marrero-Rosado et al., 2018). However, those studies did not evaluate whether the delta frequencies were localized to particular regions of the cortex as we observed. It worth noting that neuropathology associated with organophosphate (soman) toxicity correlates most closely with increased power in delta frequencies, with lesion severity occurring mostly in piriform and perirhinal cortices but also in subcortical limbic areas (McDonough et al., 1998).

Therapeutic drugs to treat organophosphate poisoning were investigated for their effects on DFP-induced EEG abnormalities. Treatments were useful in reducing the duration of abnormal EEG activity in the brain to different extents depending on the drug. Abnormal EEG activity in the delta frequencies were significantly reduced by dequalinium chloride, but not by atropine and HI6. The reduction in theta frequency was significantly reduced only after 24 h in dequalinium and HI6 treatments, but not by atropine. Theta oscillations have been associated with voluntary and exploratory behaviors in rodents and are reduced in immobile animals (Bland, 1986). A possibility is that organophosphate reduction of theta frequencies could be due to impairment of consciousness (Peter et al., 2014).

Behaviors due to organophosphate poisoning include tremors (Cannard, 2006). In this study, we also found obvious tremors in DFP treated mice. Given that there were many instances in the video-EEG records where abnormal electrographic activity did not cause tremors, or where tremors did not occur coincident with abnormal electrographic activity, it is likely that EEG activity and tremors are independent symptoms of DFP toxicity. This is consistent with other studies showing that low concentrations of DFP (2 mg/Kg) in rats caused tremors but are subthreshold for electrographic seizures (Pouliot et al., 2016). Previous studies have shown that nicotinic receptor antagonist (mecamylamine), but not muscarinic receptor antagonists (Trihexyphenidyl), eliminate organophosphate (paraoxon)-induced tremors (Iha et al., 2019) and therefore ascribe a primary role of the nicotinic receptors in organophosphate-induced tremors. In this study, we did find partial rescue of acute tremors with muscarinic antagonist atropine which would suggest that nicotinic antagonism may not be sufficient to ameliorate tremors. The discrepancy between the two studies may be because atropine has some cross-inhibition with nicotinic receptors (Zwart and Vijverberg, 1997; Parker et al., 2003; Gonzalez-Rubio et al., 2006), which would add some benefit for its use in moderating activation of both arms of the cholinergic systems. In addition, the non-specific anticholinergic dequalinium chloride reduced tremors acutely and also provided a complete elimination of tremors after 24 h while atropine did not.

Given that respiratory depression is proposed to be a major cause of organophosphate mortality (Robb and Baker, 2021), we investigated DFP and treatments on respiratory mechanics using identical dosages and timelines as the EEG studies. Similar to other studies using paraoxon, we indeed found a depression of breathing rates with DFP, although we saw a decrease rather than an increase in tidal volumes (Villa et al., 2007; Houze et al., 2008). The depression occurred both acutely and was sustained 24 h after DFP administration. The mechanism of respiratory depression has been proposed to be via central nervous system effects on muscarinic receptors since atropine (10 mg/Kg), which crosses into the brain, but not methyl-atropine, which is restricted to the periphery, ameliorated organophosphate (paraoxon) effects on respiratory mechanics in rats (Houze et al., 2008). In our study, we found that atropine (3 mg/Kg) did restore tidal volumes but not breathing rates to near control levels after 24 h. HI6 and dequalinium chloride had equivalent effects on tidal volumes as atropine but were more effective in ameliorating both acute and 24 h effects of DFP on breathing rate.

In conclusion, we found that established treatments, atropine and HI6, and the non-selective anticholinergic dequalinium chloride had distinct effects in ameliorating major CNS and respiratory symptoms of organophosphate toxicity. This is the first study to evaluate a non-selective antagonist to target both arms of the cholinergic system for organophosphate toxicity and the findings suggest either equivalent or better outcomes using this drug. Dequalinium chloride has historically been used as an anti-bacterial antiseptic, but its injections into humans remains to be tested. However, its effects on DFP-induced CNS dysfunction suggest that this compound crosses the blood-brain barrier and may be useful for other diseases where therapeutics are needed that target central cholinergic signaling.

## Data Availability Statement

The raw data supporting the conclusions of this article will be made available by the authors, without undue reservation.

## Ethics Statement

The animal study was reviewed and approved by UT Health San Antonio IACUC Committee.

## Author Contributions

VB, SG, MB-C, and RZ performed the experiments. VB, SG, RZ, and RB analyzed the data. VB, RZ, and RB prepared the manuscript. All authors had a significant contribution in its preparation and approved the submitted version.

## Conflict of Interest

The authors declare that the research was conducted in the absence of any commercial or financial relationships that could be construed as a potential conflict of interest.

## Publisher’s Note

All claims expressed in this article are solely those of the authors and do not necessarily represent those of their affiliated organizations, or those of the publisher, the editors and the reviewers. Any product that may be evaluated in this article, or claim that may be made by its manufacturer, is not guaranteed or endorsed by the publisher.
