# Supplementary figures and images for: Effects of Sublethal Organophosphate Toxicity and Anti-cholinergics on Electroencephalogram and Respiratory Mechanics in Mice
Source: Front Neurosci. 2022 May 2;16:866899. doi: 10.3389/fnins.2022.866899 (PMC9108673; doi:10.3389/fnins.2022.866899)

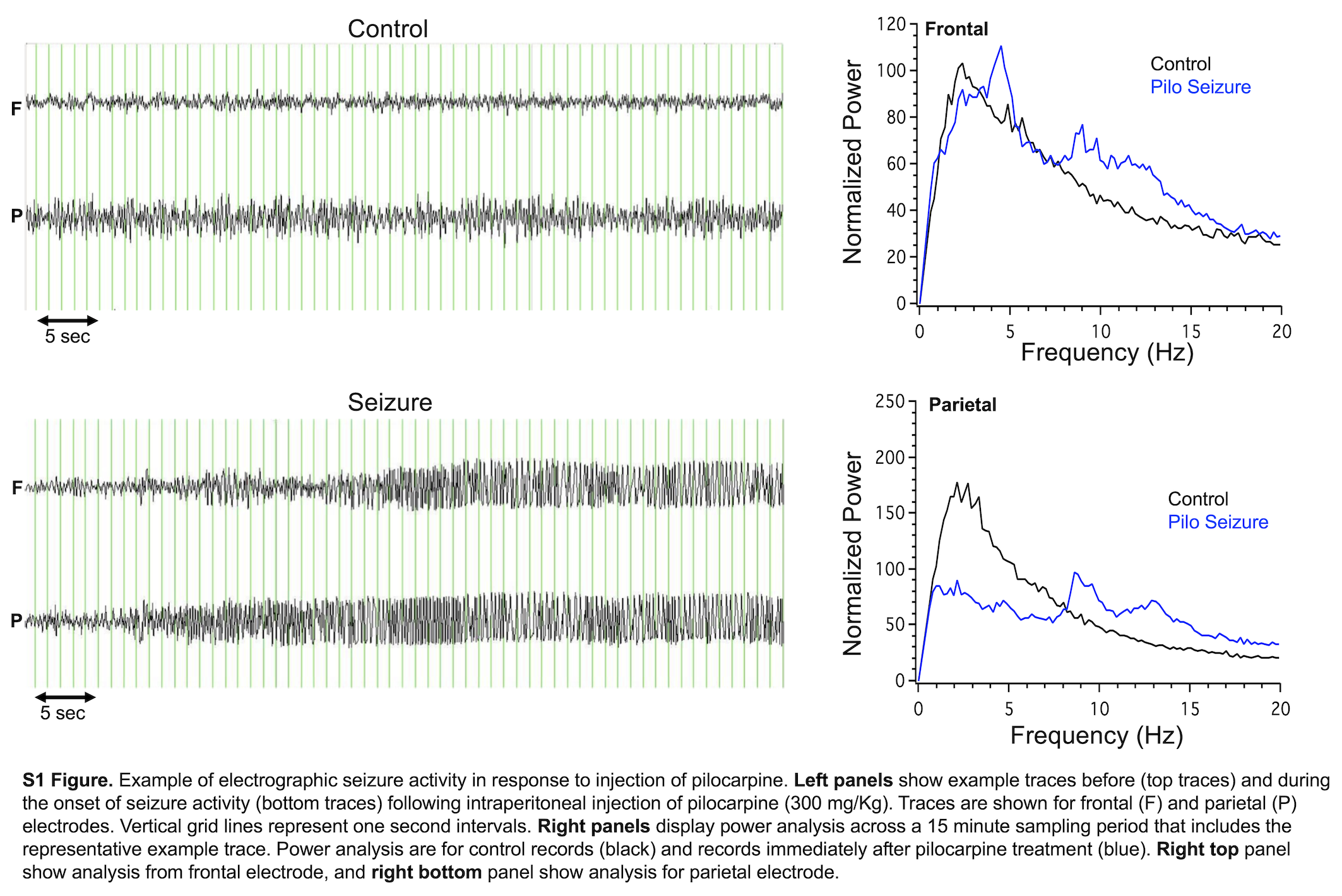

Supplement: Supplementary file 1 [file Image_1.TIFF]

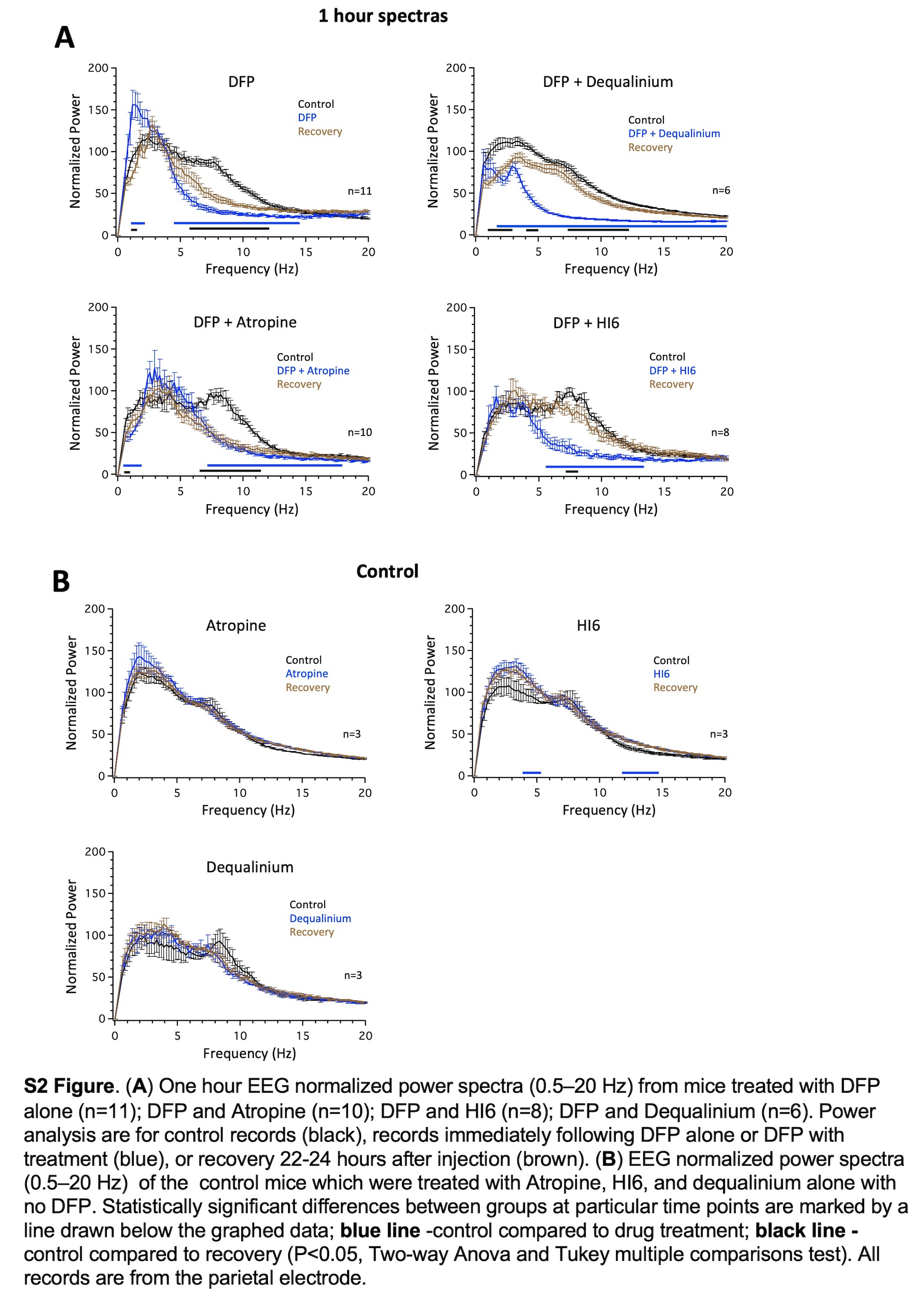

Supplement: Supplementary file 2 [file Image_2.JPEG]

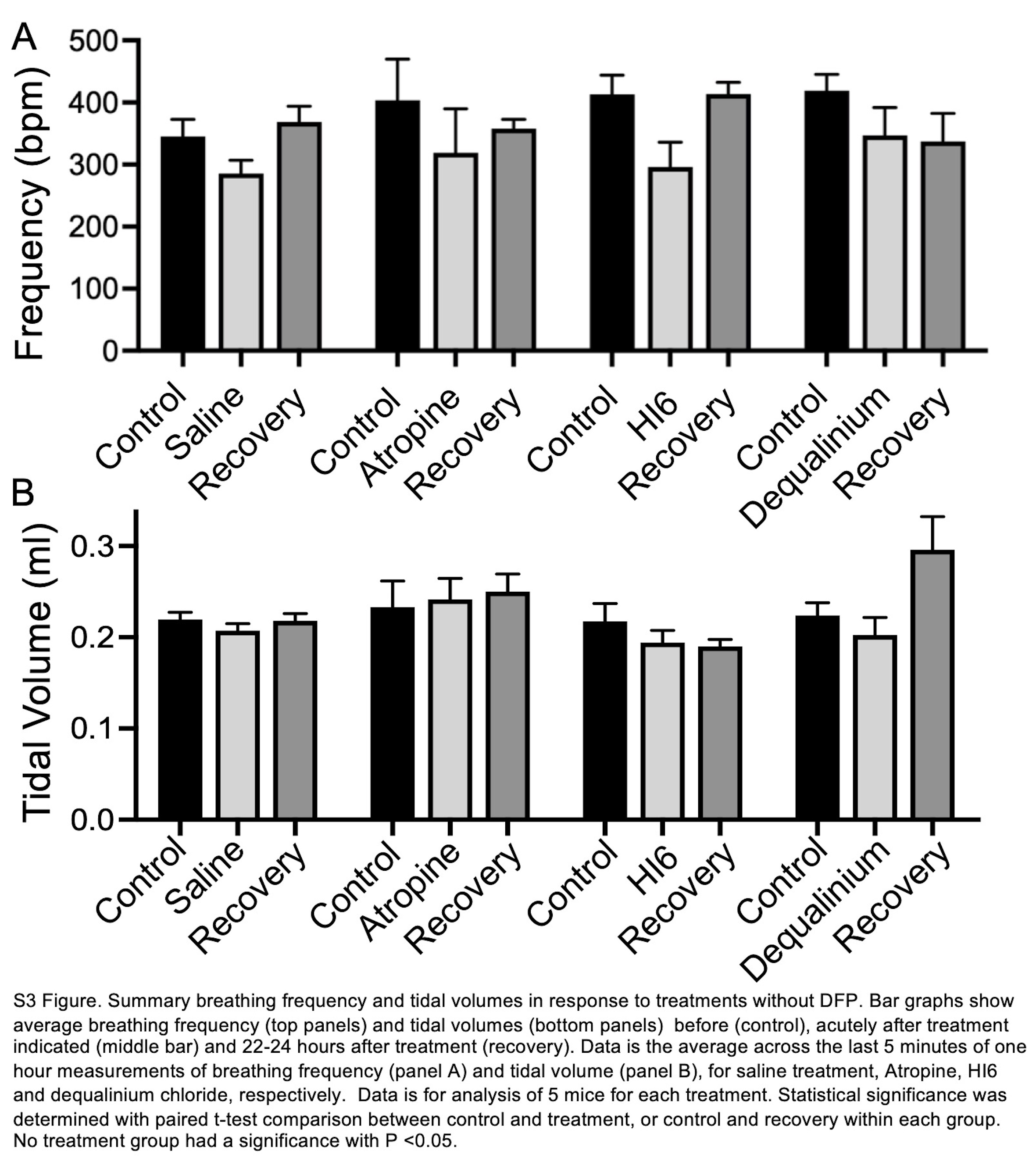

Supplement: Supplementary file 3 [file Image_3.JPEG]

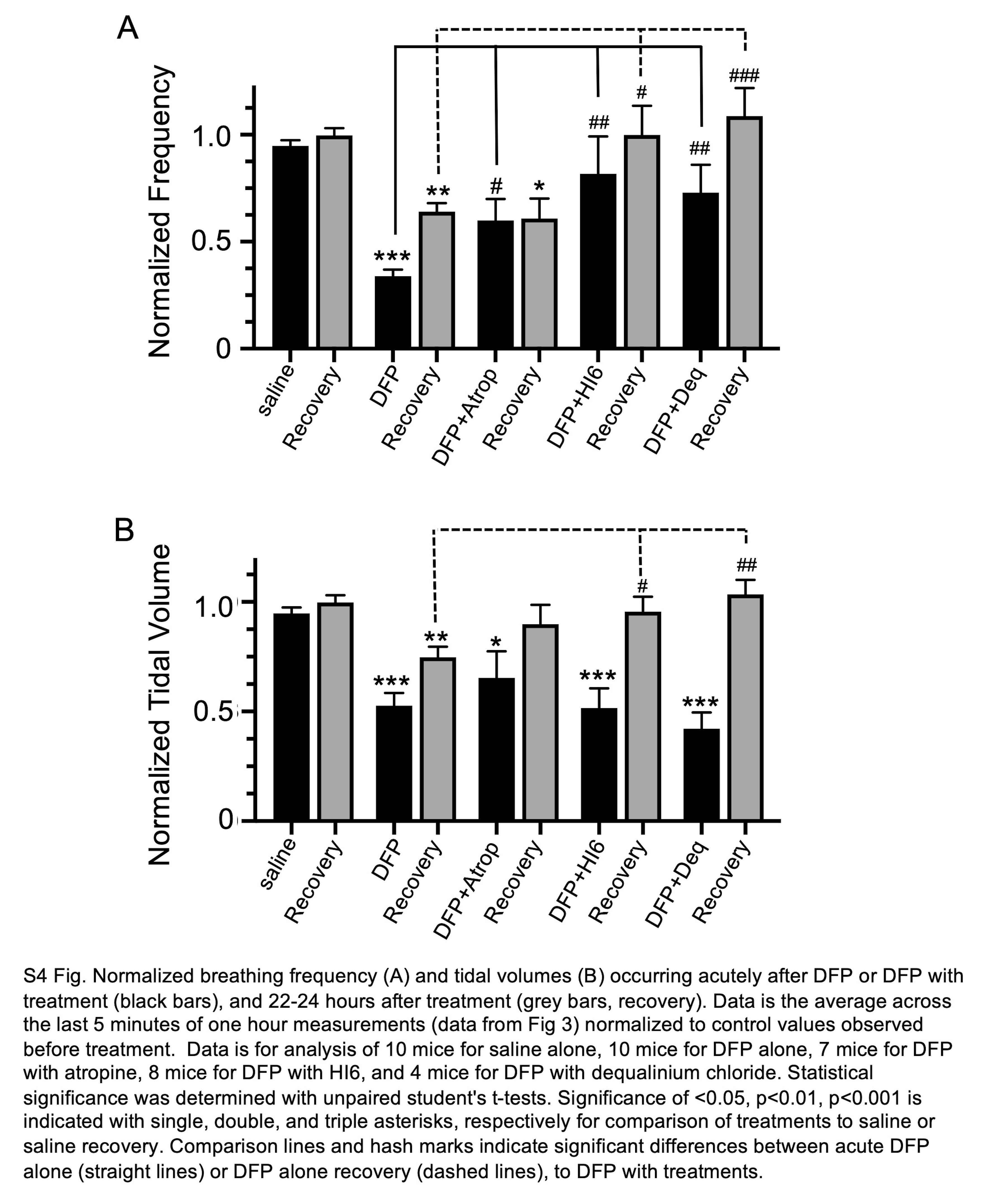

Supplement: Supplementary file 4 [file Image_4.JPEG]
